# Bone marrow lesions can be subtyped into groups with different clinical outcomes using two magnetic resonance imaging (MRI) sequences

**DOI:** 10.1186/s13075-015-0780-5

**Published:** 2015-09-27

**Authors:** Anita E. Wluka, Andrew J. Teichtahl, Rheza Maulana, Bonnie M. Liu, Yuanyuan Wang, Graham G. Giles, Richard O’Sullivan, David Findlay, Flavia M. Cicuttini

**Affiliations:** Department of Epidemiology and Preventive Medicine, School of Public Health and Preventive Medicine, Monash University, Alfred Hospital, 99 Commercial Road, Melbourne, VIC 3004 Australia; Baker IDI Heart and Diabetes Institute, 75 Commercial Road, Melbourne, VIC 3004 Australia; Cancer Epidemiology Centre, Cancer Council Victoria, Carlton, Australia and Centre for Epidemiology and Biostatistics, Melbourne School of Population and Global Health, The University of Melbourne, 207 Bouverie Street, Carlton, VIC 3010 Australia; Healthcare Imaging Services, Epworth Hospital, 89 Bridge Road, Melbourne, VIC 3121 Australia; Discipline of Orthopaedics and Trauma, University of Adelaide, North Terrace, Adelaide, SA 5005 Australia

**Keywords:** Knee, Osteoarthritis, Bone marrow lesion, Pain

## Abstract

**Introduction:**

Bone marrow lesions (BMLs) are features detected on MRI that are important in the pathogenesis of knee osteoarthritis. Since BMLs reflect heterogeneous pathologies this prospective cohort study examined whether BMLs detected using different MRI sequences are associated with distinct structural and clinical endpoints.

**Methods:**

A total of 297 community-based adults without knee pain were examined to identify BMLs visualised using three-dimensional T1-weighted gradient-echo fat-suppressed (T1-weighted sequences) fat-suppressed and fat-saturated FSE T2-weighted MRI sequences (T2-weighted sequences) at baseline. Cartilage volume was measured at baseline and follow-up, while incident knee pain was assessed at follow-up, an average of 2.3 years later.

**Results:**

At baseline, 46 BMLs were visualised in 39 participants. Of the 45 BMLs visualised on T2-weighted sequences, 34 (74 %) were also seen on T1-weighted sequences. One BML was seen on only T1-weighted sequences. Knees with BMLs visualised on both T1- and T2-weighted sequences had significantly higher medial tibial cartilage volume loss (45 mm^3^/annum, standard error of the mean (SEM) 14) than those with BMLs identified on only T2-weighted sequences (−13 mm^3^/annum SEM 19), after adjustment for age, gender and body mass index (*p* = 0.01). Incident knee pain was more likely in individuals with BMLs in the medial compartment visualised on both T1- and T2-weighted (eight participants, 53 %) compared to those with BMLs on only T2-weighted sequences (0 %) or no BMLs (76 participants, 31 %, *p* = 0.02).

**Conclusions:**

BMLs present on both T1- and T2-weighted MRI sequences were associated with increased medial tibial cartilage loss and incident knee pain compared with those BMLs seen only on T2-weighted sequences. This suggests that combining different MRI sequences may provide more informative targets in the prevention and treatment of knee osteoarthritis.

## Introduction

Bone marrow lesions (BMLs) are important in the pathogenesis of knee osteoarthritis (OA). Both in those with and without symptomatic knee OA, their presence has been linked to knee pain [1, 2], increased cartilage volume loss [3, 4], radiographic progression [5] and joint replacement [6, 7]. BMLs are ill-defined hyperintensities on magnetic resonance imaging (MRI), and have been described using a variety of different sequences, including short inversion time inversion recovery (STIR) images, T2-weighted, fat-suppressed sequence and T1-weighted sequence [7, 8]. Although each of the different MRI sequences captures different tissue properties [9], BMLs have been considered a homogeneous group in epidemiological studies. Utilising different MRI sequences to characterise BMLs may offer new insights into the composition and clinical characteristics of BMLs.

The underlying pathology of BMLs has not been well characterised. Bone within BMLs identified using T2-weighted fat-suppressed images was shown to be sclerotic but less well mineralised than adjacent unaffected bone [10]. In another study, BMLs identified by images obtained using STIR, T1- and T2-weighted sequences showed that affected tissue, rather than being oedematous, as was initially proposed [5], consisted of bone necrosis, fibrosis and bone marrow necrosis, with little oedema [9]. In that study of 16 patients with OA, Zanetti et al. began to differentiate the underlying histology of BMLs visualised by differing combinations of STIR, T1- and T2-weighted sequences. At the lumbar spine, use of data obtained using two different sequences has been shown to better discriminate distinct vertebral body marrow and endplate lesions (Modic change) [11], each with characteristic histological and clinical correlates [11–20].

Whether BMLs at the knee identified using different sequences have similar clinical correlates is not clear. However, while T2-weighted hyperintensities may be able to identify more BMLs [21–23] compared to T1-weighted sequences, BMLs identified from either T1- or T2-weighted images predict joint replacement surgery among people with knee OA [6, 24]. Importantly, whether BMLs identified by different MRI sequences are differentially related to clinically relevant outcomes such as cartilage loss and the development of pain has not been examined.

To do so, we examined the prevalence of BMLs identified in the knees of asymptomatic adults using both T1- (three-dimensional T1-weighted spoiled gradient echo (SPGR) fat-suppressed) and T2-weighted (T2-weighted fat-saturated) sequences. We investigated whether BMLs identified from these T1-, T2- or both T1- and T2-weighted images related differentially to the rate of cartilage volume loss and the development of knee pain in adults without diagnosed knee OA.

## Methods

### Participants

The study was conducted within the Melbourne Collaborative Cohort Study (MCCS), a prospective cohort study of 41,514 people, as described previously [25]. We invited subjects who attended the first year of the second follow-up of the MCCS (2003–2004), provided they were aged between 50 and 79 years and did not meet a clinical diagnosis of knee OA as defined by American College of Rheumatology criteria [26]. Participants were excluded if they indicated a history of knee pain lasting for > 24 hours in the last 5 years; a previous knee injury requiring non-weight-bearing treatment for > 24 hours or surgery (including arthroscopy); a history of any arthritis diagnosed by a medical practitioner; or any contraindication to MRI. Quota sampling was used, whereby recruitment ceased when our target of approximately 300 subjects was reached. A follow-up MRI was performed between 2006 and 2007, with an average of 2.3 years having elapsed since the initial imaging study. The study was approved by the Human Research Ethics Committees of The Cancer Council Victoria and Monash University. All participants gave written informed consent.

### Anthropometric measures

Height (cm) was measured at baseline using a stadiometer with footwear removed. Weight (kg) was measured with bulky clothing removed at the time of baseline MRI. Body mass index (BMI) was calculated from these data [weight (kg)/height^2^ (m^2^)].

### MRI image acquisition

In 2003–2004 and again in 2006–2007, the dominant knee (defined as the lower limb the subject used to kick a ball) of each subject was imaged using MRI. At each visit, knees were imaged on a 1.5-T whole body magnetic resonance unit (Philips 1.5 Tesla Intera; Philips Medical Systems, Eindhoven, The Netherlands) using a commercial transmit-receive extremity coil. The following sequences and parameters were used: a T1-weighted fat-suppressed three-dimensional gradient recall acquisition in the steady state; flip angle 55 degrees; repetition time 58 msec; echo time 12 msec; field of view 16 cm; 60 partitions; 512 × 512 matrix; one acquisition time 11 minutes 56 seconds. Sagittal images were obtained at a partition thickness of 1.5 mm and an in-plate resolution of 0.31 × 0.31 mm (512 × 512 pixels). These sequences will be referred to as T1-weighted sequences hereafter throughout the manuscript. In addition, a coronal T2-weighted fat-saturated acquisition (T2-weighted fat-saturated), repetition time 3500–3800 msec, echo time 50 msec, with a slice thickness of 3.0 mm, a 1.0 mm interslice gap, 1 excitation, a field of view of 13 cm, and a matrix of 256 × 192 was also obtained [27]. The acquisition time for the T2-weighted fat-saturated sequence was 4 minutes 46 seconds. The echo train length was 7. These sequences will be referred to as T2-weighted sequences throughout the manuscript. All MRI images were read by a clinical radiologist with musculoskeletal MRI expertise to identify the presence of any clinically significant abnormalities.

### MRI related measures

**Fig. 1:**
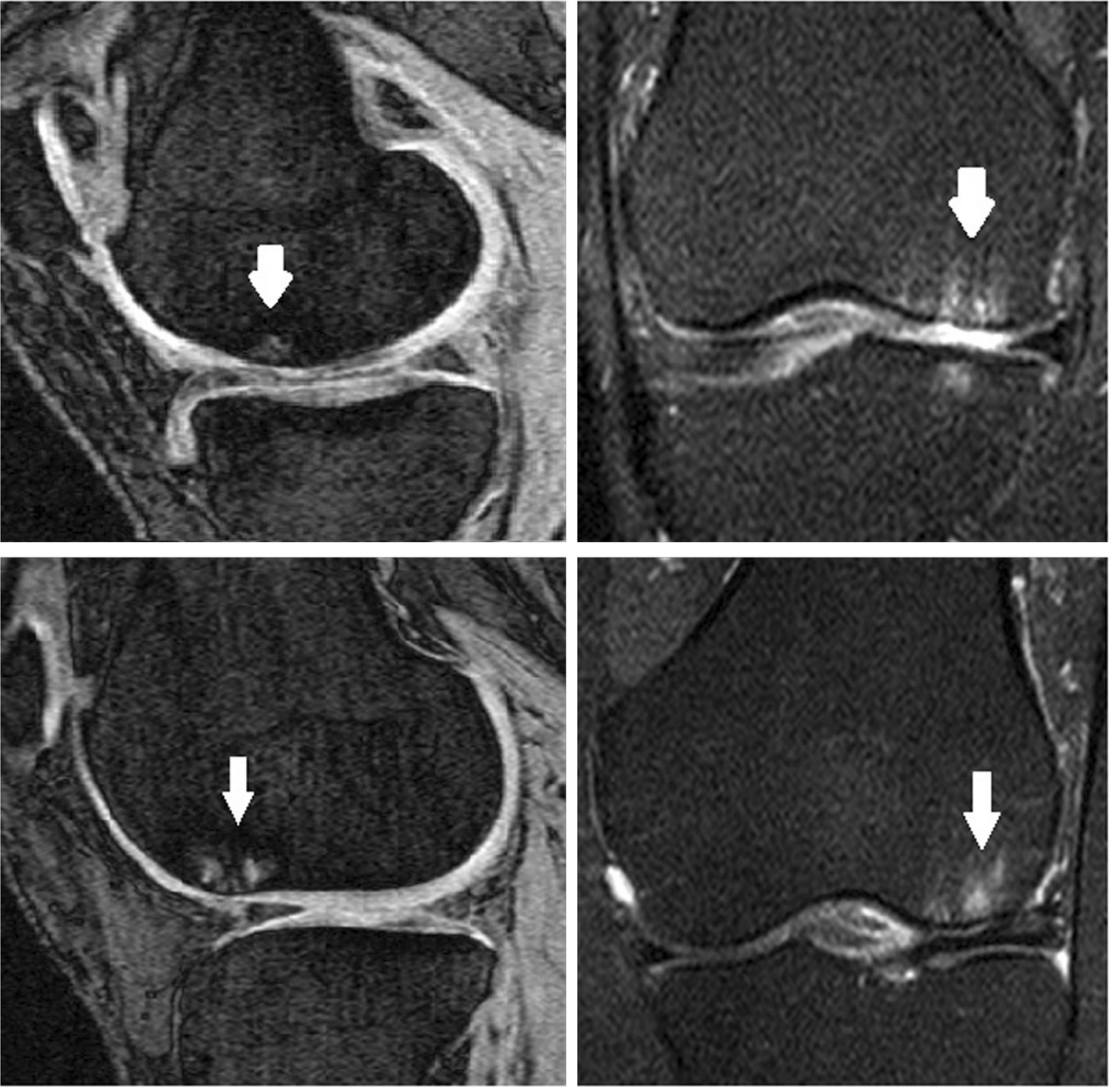
Two BMLs visualised on both T1- and T2-weighted sequences are shown, one on each row. The images on the *left*, obtained in the sagittal plane, show the BMLs identified using T1-weighted sequences. The images on the *right*, obtained in the coronal plane, show the same BMLs imaged using the T2-weighted sequences. *BML* bone marrow lesion

BMLs were defined as areas of increased signal intensity immediately underlying subchondral bone in either the medial or lateral distal femur or proximal tibia in either imaging sequences [24, 28]. Two trained observers, who were blinded to patient characteristics, as well as image sequence, together assessed the presence of BMLs for each subject. The presence or absence of BMLs was determined. Figure 1 demonstrates two BMLs seen using both sequences. The reproducibility for the presence of BMLs was assessed using 60 randomly selected knee MRIs (к value 0.88, *p* < 0.001) [29].

*Cartilage volume* was determined by image processing on an independent workstation using the Osiris software. Because femoral cartilage volume [30], and change in femoral cartilage volume [31] is less reproducible and highly correlated with tibial cartilage and change in tibial cartilage volume, tibial cartilage volume was assessed. The volumes of the individual cartilage plates (medial and lateral tibial) were measured from the total volume by manually drawing disarticulation contours around the cartilage boundaries on each section. The volume of the particular cartilage plate was determined by summing the pertinent voxels within the resultant binary volume. A trained observer read each MRI. Independent measures of volume were made in a blinded fashion by a second trained observer. The coefficients of variation for the measurement of cartilage volume at the medial and lateral tibia were 2.1 % and 2.2 % respectively [32]. Annual change in volume was calculated between 2003 and 2004 and 2006 and 2007 using the following equation: (baseline to follow-up)/time between scans.

### Knee pain

All participants were pain free at the beginning of the study. At the time of the second MRI they were asked “have you had any pain in your knee in the past 12 months, yes or no?” to define incident knee pain, as described previously [2].

### Statistical analyses

Differences between participants in the three groups (i) participants with no BMLs, (ii) participants with BMLs visualised using T2-weighted sequence only and (iii) participants with BMLs visualised using both T1- and T2-weighted sequences were described using analysis of variance for continuous variables and chi-square test for dichotomous variables. Estimated marginal means were used to compare differences between groups for baseline cartilage volume and annual percentage change in cartilage, with pairwise comparisons made using the F test. Where a person had more than one BML in that compartment, only the largest BML visualised using T2-weighted images was used. Chi-square tests were used to determine any between-group differences in the frequency of incident knee pain. *P* values of less than 0.05 were considered to be statistically significant. All analyses were performed using the SPSS statistical package (standard version 20.0, IBM Corp., Armonk, NY, USA).

With a sample size of 271, this study had 80 % power to detect a correlation as low as 0.20 between BMLs and annual change in cartilage volume (alpha error 0.05, two-sided significance).

## Results and discussion

Comparisons of the participant characteristics according to the presence of BMLs on T2-weighted images, T1- and T2-weighted images or absence of BMLs at baseline are shown in Table 1. A single participant had a BML that was visualised only on the T1-weighted images in the medial femoral condyle (characteristics not shown). There were no significant differences in the baseline characteristics of participants according to their grouping (no BML present, BMLs visualised on T2-weighted images only and BMLs visualised on T1- and T2-weighted imaging). Of the other 296 pain-free participants imaged at baseline, 270 (92 %) completed the study. Apart from those lost to follow-up having higher BMI (*p* = 0.01) than those who completed the study, there were no significant differences between people who did and did not complete follow-up (data not shown).

**Table 1:** Baseline participant characteristics according to the presence of any BML being present^1^

	No BML	BML on T2 only	BML on T1 and T2	*p* value^2^
	*N* = 258	*N* = 9	*N* = 29	
Age (years)	57.7 (5.2)	59.8 (7.6)	59.9 (6.0)	0.06
Gender, n (%) female	159 (62)	7 (78)	20 (69)	0.48^3^
BMI (kgm^−2^)	25.7 (4.1)	27.0 (4.0)	27.5 (5.5)	0.09
Baseline tibial cartilage volume (mm^3^)				
Medial	1695 (532)	1609 (382)	1807 (554)	0.49
Lateral	2026 (648)	1994 (724)	2012 (610)	0.98

Results expressed as mean (standard deviation) or number (%)

*BML* bone marrow lesion, *BMI* body mass index

^1^One participant had a BML present only on T1-weighted sequences, and was not included

^2^ *p* value expressed as F test – pairwise comparisons among the unadjusted means

^3^Chi-square test

In total, 46 BMLs were seen in 39 participants, with 33 participants having a single BML, five having two BMLs present and one having three BMLs. Of these, 45 BMLs (98 %) were visible on images obtained using T2-weighted sequences, including 34 BMLs (74 %) also visible on T1-weighted fat-suppressed images. A single BML was visible on T1-weighted images only. Thus 11 (24 %) were visible only in T2-weighted images. The sites in which BML were identified using T2-weighted sequences are shown in Table 2. Four participants had more than one BML present in the medial compartment. The volume of BMLs visualised using T1- and T2-weighted sequences are also shown, as are the ratios between the volumes measured from T1- and T2-weighted sequences. Whilst BML visualised on T2-weighted sequences tended to be larger than those visualised on T1-weighted sequences, this was not universal.

**Table 2:** Site and volume (mm^3^) of BML according to whether visualised by T2 fat-saturated images only or both T1- and T2-weighted images

	BML seen in T2 only		BML seen in T1 and T2			
	n	T2 volume^1^	n	T2 volume^1^	T1 volume^1^	% T2 volume^2^
Medial compartment						
Femoral	4	214 (66–543)	12	580 (54–3160)	56 (11–292)	17 % (4–51 %)
Tibial	5	127 (54–328)	6	287(46–616)	163 (14–601)	63 % (5–148 %)
Lateral compartment						
Femoral	0	--	8	1407(145–3957)	271 (12–1002)	12 % (6–47 %)
Tibial	2	289 (106–472)	8	530 (89–1604)	107 (14–335)	22 % (9–40 %)

*BML* bone marrow lesion

^1^Mean and range of BML volume identified by T2- or T1-weighted sequences, respectively

^2^BML volume measured from T1-weighted sequences compared to BML volume measured from T2-weighted sequences, expressed as a percentage

We examined whether cartilage volume and annual percentage change in cartilage volume at the knee differed in those individuals without BMLs, compared to those individuals with BMLs visible on the differently weighted MRI sequences (Table 3). Of the 271 participants who completed follow-up, one participant had a BML visualised on T1-weighted images only, 37 participants had BMLs visualised on T2-weighted images, with 29 (78 %) also visualised on T1-weighted images. Baseline medial and lateral cartilage volume was similar in the three groups, and also after adjusting for baseline age, gender and BMI. Annual loss of medial tibial cartilage tended to be different between the three groups (*p* = 0.07). After adjusting for age, gender and BMI, annual loss of medial tibial cartilage was significantly greater in those where a BML was visualised using T1- and T2-weighted imaging (45 mm^3^/annum, standard error of the mean (SEM) 14) compared to those with BML present in T2-weighted images only (−13 mm^3^/annum, SEM 19) and those with no BML (22 mm^3^/annum, SEM 3) (*p* = 0.04). The difference in cartilage loss in those with a BML seen in T1- and T2-weighted images compared to those with a BML seen in T2-weighted images only was significant (57 mm^3^/annum, SEM 23, *p* = 0.01). Annual loss of lateral tibial cartilage volume was significantly greater in participants where a BML was visualised on T1- and T2-weighted images (55 mm^3^/annum, SEM 3) than when no BML was seen (22 mm^3^/annum) (*p* = 0.01 for difference between these two groups).

**Table 3:** Relationship between the presence of BML according to sequence at baseline and tibial cartilage volume^1^

	No BML	BML in T2 only	BML in T1 and T2	*p* value^4^
Baseline cartilage volume (mm^3^)^2^				
Medial compartment				
	*n* = 273	*n* = 7	*n* = 16	
Medial	1705 (32)	1552 (200)	1743 (133)	0.72
Medial, adjusted^2^	1698 (22)	1722 (139)	1809 (95)	0.52
Lateral compartment				
	*n* = 278	*n* = 2	*n* = 16	
Lateral	2014 (39)	2462 (456)	2176 (161)	0.33^5^
Lateral, adjusted^2^	2020 (28)	2356 (323)	2137 (117)	0.34^5^
Annual change in cartilage volume (mm^3^)				
Medial compartment				
	*n* = 248	*n* = 7	*n* = 15	
Medial	22 (3)	−11 (19)	42 (13)	0.07
Medial, adjusted^3^	22 (3)	−13 (19)	45 (14)	0.04
Lateral compartment				
	*n* = 254	*n* = 2	*n* = 14	
Lateral	22 (3)	70 (36)	55 (13)	0.02^5^
Lateral, adjusted^3^	22 (3)	69 (36)	55 (14)	0.02^5^

*BML* bone marrow lesion

^1^One participant had a BML present only on T1-weighted sequences, and was not included

^2^Estimated marginal means (standard error of the mean, SEM) used to compare groups adjusted for baseline age, gender, body mass index (BMI)

^3^Estimated marginal means (SEM) used to compare groups adjusted for baseline age, gender and BMI. Annual change is expressed such that a negative value represents a gain in cartilage volume

^4^ *p* value for F test, pairwise comparisons among the estimated marginal means across all groups, except where indicated

^5^For lateral compartment, the comparison was between those with no BML at baseline and those with BML visible using both T1- and T2-weighted images, as *n* = 2 for BML visible using T2-weighted images only

We examined whether the risk of incident knee pain differed according to the presence of BMLs visualised using the different sequences at baseline (Table 4). Eighty-five participants developed knee pain during the study period. Participants with BML in the medial compartment visualised by both T1- and T2-weighted sequences were more likely to develop pain than those with no BML at baseline or a BML visualised using T2-weighted images only (chi-square, *p* = 0.04). Those with a BML visualised using both T1- and T2-weighted sequences were more likely to develop pain than those with BML visualised only on T2-weighted images (Fisher’s exact test, *p* = 0.02) Incident knee pain was not associated with the presence of BMLs in the lateral compartment (*p* = 0.79).

**Table 4:** Participants who developed pain according to the presence of baseline BMLs

	Number at baseline	Pain developed	*p* value^1^
Medial compartment			
No BML	248	76 (31 %)	0.04
BML in T1	1	1 (100 %)^2^	
BML in T2	7	0 (0 %)	
BML in T1 and T2	15	8 (54 %)	
Lateral compartment			
No BML	279	79 (31 %)	0.79
BML in T2	2	1 (50 %)	
BML in T1 and T2	16	5 (36 %)	

*BML* bone marrow lesion

^1^Chi-square test, likelihood ratio

^2^Data excluded from analysis

This study examined and characterised clinical and structural correlates of BMLs identified using two different MRI sequences in asymptomatic adults. BMLs visualised using both T1- and T2-weighted sequences were the most common. When these were compared to BMLs visualised only using T2-weighted sequences, they were associated with greater medial tibial cartilage volume loss and more frequent incident knee pain over 2 years. This suggests that using information from multiple MRI sequences to characterise BMLs may provide more prognostic information than using a single sequence alone.

Different MRI sequences have been developed to identify and better delineate tissue characteristics and pathologies. At the knee, when BMLs were initially described, they were assumed to represent bone oedema [5]. Histological studies failed to corroborate this, and these abnormalities have subsequently been referred to BMLs rather than bone oedema [9, 10]. Sequences deemed to be water sensitive, such as fat-suppressed T2-weighted (FST2w), proton density-weighted (PDw), intermediate-weighted fat-saturated (IWFS)-fast spin echo (FSE) or STIR, have been used to determine the presence of BMLs in epidemiological studies, although BMLs are visible using other sequences such as T1-weighted images [7]. Nevertheless, these water-sensitive sequences differ in their imaging of BMLs, with IWFS-FSE and dual-echo steady state (DESS) imaging sequences having been shown to both identify BMLs in 65 % of cases, but to be discordant in identifying BMLs in 36 % of cases [8]. In the current study, we examined T1- and T2-weighted sequences as described, and have demonstrated differences in their identification of BMLs also. These differences may represent variation in underlying histology of what has been designated a BML [9]. It has previously been shown that by categorising tissue according to its imaging characteristics using STIR, T1- and T2-weighted sequences, differences in tissue histology could be predicted [9]. That there was no consistent relationship between the volume of BMLs visualised on the T1- and T2-weighted sequences used in this study supports this contention. This variation in the characteristics of the underlying bone may account for differences in the relationship between the presence of a BML and subsequent cartilage loss and clinical disease.

BMLs visualised by both T1- and T2-weighted sequences were associated with significantly greater cartilage volume loss compared to knees that had BMLs present only in T2-weighted images. This is the first study to examine this question. Previously, we have shown BMLs identified using T1-weighted images to be associated with an increased risk of joint replacement [7]. Others have shown BMLs identified by three-dimensional spoiled gradient-recalled acquisitions with fat suppression [33] or three-dimensional fast imaging steady state precession acquisitions with water excitation [6], more closely related to the T2-weighted images used in the current study, to be predictive of arthroplasty [6]. Thus it is not possible to compare the strength of these relationships using the available literature. Although there was a tendency for those with BMLs present in T2-weighted images only to show increased medial tibial cartilage volume, this gain was not significant (*p* = 0.07). It is important to consider this in context, as this cohort was initially pain free and did not have clinical OA. In animal models, one of the first changes observed in cartilage is swelling [34]. It is possible that in this initially asymptomatic cohort, BMLs apparent on T2-weighted images only identified people at risk for early cartilage damage, with cartilage swelling. Whether the results that have shown BMLs visualised using T2-weighted sequences to be associated with clinical endpoints including knee pain [35, 36], cartilage loss [24, 29, 37, 38], radiographic progression [5] and knee arthroplasty [6] would be strengthened by further stratification by the presence of BMLs visualised using T1-weighted imaging is unknown. At the back, there is evidence, albeit limited, that Modic type 1 and 2 changes have different relationships to low back pain, suggesting that use of multiple sequences may enable better phenotyping of musculoskeletal conditions [15].

Although this study has demonstrated that BMLs identified by different MRI-weighted sequencing predict people at risk for developing accelerated medial tibial cartilage volume loss and incident knee pain, the mechanism for this remains unclear. Different components of BMLs detected by different MRI sequences detect varied histology [9]. This situation is potentially analogous to what has been described at the lumbar spine. Modic changes, which are vertebral body marrow and endplate lesions seen on MRI, have been classified according to their characteristic appearance on T1 (FSE two-dimensional T1-weighted sequences) and T2-weighed images (FSE two-dimensional T2-weighted images) [11], each with different histological associations. Modic type 1 change histologically represents bone marrow oedema and inflammation and are strongly associated with low back pain [12–15, 17] and instability [14, 16]. Modic type 2 change represents marrow ischaemia and the conversion of normal red haemopoietic bone marrow into yellow fatty marrow [11] and are more frequent among individuals with degenerative disc disease [14, 18, 19]. Modic type 3 change is rare and represents subchondral bone sclerosis and microfracture [20]. Although the current data provide evidence that different MRI sequencing of BMLs are associated with different structural and symptom endpoints, how the differences in BMLs relate to histological features is not fully characterised. What is, however, interesting from the analogy between BMLs at the knee and Modic type 2 change at the lumbar spine is that both abnormalities are associated with deleterious cartilage changes. That is, Modic type 2 change, which is hyperintense on both the described T1- and T2-weighted images, is associated with intervertebral disc degeneration [14, 18, 19], while knee BMLs visualised by both the studied sequences are more closely associated with accelerated medial tibial cartilage volume loss than those seen in only the T2-weighted sequences examined. Although the intervertebral disc comprises fibrocartilage, while articular cartilage at the knee is hyaline, these commonalities might suggest a similar underlying pathophysiology. Histological differences among BMLs detected by varying MRI sequences may be a key determinant of why certain lesions predate accelerated cartilage loss and the development of incident knee pain.

This study has several limitations. We have examined a large community-based population of adults without diagnosed knee OA and thus our results may not be generalisable to those with knee OA. However, the structural changes that we describe occur on a continuum from the healthy to the diseased knee. As we examined an asymptomatic population at baseline, BMLs were not common, so that their relatively small numbers gave us limited power to assess their correlates. Despite this, we were able to demonstrate significant differences between those with BMLs visualised only on T2-weighted images and those with BMLs visualised on both T1- and T2-weighted images. Examination of a larger population, incorporating people with knee pain may help to address this issue.

## Conclusions

This study examined and characterised BMLs detected by different MRI-weighted sequences. BMLs visualised from both T1- and T2-weighted images were most common and when compared to BMLs seen in only T2-weighted images, were associated with greater medial tibial cartilage volume loss and more incident knee pain. Determining the underlying histology of BMLs visualised using different sequences may allow us to use MRI as a proxy for histology, thus enabling a better understanding of the pathogenesis of OA and optimise targeting of therapies aimed at treatment and prevention of knee OA.

## Abbreviations

BMI: body mass index
BML: bone marrow lesion
DESS: dual-echo steady state
FSE: fast spin echo
IWFS-FSE: intermediate-weighted fat-saturated
MRI: magnetic resonance imaging
OA: osteoarthritis
PDw: proton density-weighted
SEM: standard error of the mean
SPGR: spoiled gradient echo
STIR: short inversion time inversion recovery
